# A prospective, randomised study of the effect of fixation sutures during phacotrabeculectomy on intraocular pressure and incidence of ptosis

**DOI:** 10.1038/s41598-020-79635-x

**Published:** 2021-01-12

**Authors:** Joanna Konopińska, Łukasz Lisowski, Zofia Mariak, Małgorzata Wojnar, Iwona Obuchowska, Marek Rękas

**Affiliations:** 1 Department of Ophthalmology, Medical University, Białystok, Poland; 2 Department of Ophthalmology, Wojskowy Instytut Medyczny, Warsaw, Poland

**Keywords:** Diseases, Health care, Medical research

## Abstract

We investigated the effects of different intraoperative eyeball fixation techniques (superior rectus muscle suture [MS] and traction suture at the corneal limbus [CS]), on intraocular pressure (IOP) and the incidence of ptosis after phacotrabeculectomy. Forty-one eyes with different glaucoma types which qualified for phacotrabeculectomy were included. Twenty-three and eighteen patients were included in the CS and MS groups, respectively. The IOP, best-corrected visual acuity (BCVA), and margin reflex distance were assessed preoperatively and 3, 6, and 12 months post-operatively. Preoperatively, the mean IOPs (± standard deviation) in the CS and MS groups were 23.6 ± 7.3 mmHg and 24.3 ± 6.6 mmHg (*p* > 0.05), respectively. At 3 and 6 months post-surgery, the mean IOPs were significantly lower in the CS group than in the MS group: 13.9 ± 3.0 mmHg vs. 17.7 ± 3.5 mmHg (*p* = 0.001), and 13.9 ± 4.9 mmHg vs. 17.2 ± 3.5 mmHg (*p* = 0.005), respectively (mean difference: 3.9, 95% confidence interval 1.7–6.1). At 12 months, the mean postoperative IOPs were 15.2 ± 3.5 mmHg and 14.9 ± 3.6 mmHg in the CS and MS groups, respectively (*p* > 0.05). At 6 months, the BCVAs were 0.91 ± 0.15 and 0.71 ± 0.3 (*p* = 0.029) in the CS and MS groups, respectively; BCVAs were 0.91 ± 0.15 and 0.71 ± 0.3 (*p* = 0.029) in the CS and MS groups, respectively; the difference was non-significant 12 months post-surgery (0.78 ± 0.32 vs. 0.74 ± 0.30, *p* = 0.553). Postoperative ptosis was observed in 4 (17%) and zero patients in the CS and MS groups, respectively, but the difference was not statistically significant (*p* = 0.118). The study was not powered sufficiently to detect statistically significant changes in exploratory endpoints. The study was not powered sufficiently to detect statistically significant differences between groups in exploratory endpoints.

## Introduction

Trabeculectomy is still the gold standard in glaucoma surgery. The effectiveness of this technique, either as a single procedure or combined with phacoemulsification (phacotrabeculectomy), is well known and documented in the literature^1–5^. Both procedures are known to have a similar hypotensive efficacy of 35.2–47.9%, and surgical success rate reaching up to 61.2–84.5%. However, despite surgical treatment, the target IOP is not achieved in 15–38.8% of patients. This results primarily from the scarring and fibrosis of the filtering bleb^6^. The high proportion of phacotrabeculectomy therapeutic failures stimulated research on additional factors contributing to the greater effectiveness of this procedure^6^.

In 2016, Li et al. showed a higher hypotensive efficacy of peripheral lamellar corneal traction suture than superior rectus traction suture in patients undergoing trabeculectomy. The qualified surgical success rates for the two techniques were 94.4% and 88.3%, respectively^7^. As hypothesized by the authors of that study, the traction suture applied throughout the conjunctiva and superior rectus muscle might promote healing, stimulating fibroblasts due to the presence of haemorrhage and tissue damage adjacent to the surgical field.

On the other hand, postoperative ptosis is a common complication of ophthalmic surgeries, and as such, it has been well documented in the literature; however, there is a lack of prospective studies on this subject^8–12^. Ptosis is reported to develop in 5–14% of patients after cataract surgeries^8^ and in 8–19% of patients who underwent trabeculectomy one month earlier. This complication may be related to the type of anaesthesia and duration of the procedure^13^. The occurrence of ptosis that persisted longer than six months was also attributed to the application of superior rectus traction sutures^10^. It constitutes a significant problem in glaucoma patients as it may hinder the measurement of the IOP with an applanation tonometer, cause postoperative astigmatism, imitate visual field defects, and negatively affect patients’ psychological status.

The aim of this prospective, randomized trial was to verify if the type of intraoperative eyeball fixation influences the hypotensive efficacy of phacotrabeculectomy, and if it affects the occurrence of postoperative ptosis.

## Methods

The study protocol was compliant with the World Medical Association’s Declaration of Helsinki and satisfied the good clinical practice and good manufacturing practice requirements for the conduct of clinical trials in the European Union. The protocol was approved by the local Bioethics Committee at the Medical University of Bialystok under the number R-I-002/443/2014^3^. The study protocol was registered on www.clinicaltrials.gov under the number NCT03797846 on 09/01/2019.

The conception of the study was similar to what we described elsewhere^3^. Patients were eligible for the surgery if they presented with glaucoma and concomitant cataract (NC1, NC2) classified according to the Lens Opacities Classification System III scale. Subjects with primary open angle glaucoma (POAG), pseudoexfoliative glaucoma (PXG), and pigmentary glaucoma (PG), who did not achieve IOP control (< 21 mmHg) despite maximum IOP-lowering therapy, qualified for the procedure. Other inclusion criteria were a documented progression of visual field defects, substantial circadian fluctuations of the IOP, lack of compliance with the IOP-lowering therapy, and allergy to topical agents. Written informed consent to participate in the study for at least 12 months was sought from all patients. The exclusion criteria were a lack of informed consent, history of previous ophthalmic surgeries, narrow-angle or closed-angle glaucoma, secondary inflammatory or traumatic glaucoma, and chronic diseases of the cornea, optic nerve, or retina.

The study included 41 eyes which underwent two-site phacoemulsification combined with trabeculectomy. The patients were randomized to one of the two groups differing in terms of the intraoperative eyeball fixation technique: traction suture at the corneal limbus (CS group, 23 eyes) or superior rectus muscle suture (MS group, 18 eyes). Subjects were allocated to a group by the toss of a coin. Data were collected at the Department of Ophthalmology Medical University of Białystok, Poland. Implementation of groups (allocation, enrollment of participants, and assigning) was conducted by an operator (J.K). Recruitment took place during January 2018, and patients were followed up from January 2018 to January 2019. At the beginning the follow-up period was supposed to be 6 months, however after obtaining preliminary results the follow-up period was extended extended for another 6 months (12 months total).

### Preoperative assessment

During qualification for the surgery, detailed information about previous pharmacotherapy and surgical treatment was obtained from all patients^3^. Before the surgery, all patients underwent routine measurements of IOP, BCVA (with the Snellen chart), the examination of the anterior and posterior segment of the eye, gonioscopy, and a visual field test (Humphrey, SITA Standard 30–2). Preoperative IOP was measured following the principles of the Advanced Glaucoma Intervention Study, with a Goldmann applanation tonometer on a slit-lamp biomicroscope. The IOP measurements were taken at the same time of the day, between 8:00 AM and 10:00 AM. The intraocular lens power was calculated from the SRKT formula, using the IOL Master 700 biometry device (Carl Zeiss Meditec).

Secondary outcome measures were the palpebral fissure parameters: marginal reflex distance (MRD), upper eyelid contour (UEC), and superior rectus muscle function (SRMF) were measured in each patient. All parameters were always determined for both eyes by two independent investigators, which were then averaged. MRD was measured under standardized lighting conditions, with a handheld ruler and penlight, as described elsewhere^14^. Postoperative ptosis was defined as a reduction of MRD by at least 2 mm compared with the measurement obtained before surgery. UEC was measured as a straight line connecting the lower and upper eyelid margins and crossing the pupil during a forward gaze, as described by Akaishi et al.^15^. SRMF was measured as a difference in the elevation of the upper eyelid margin during a maximum downward and upward gaze^16^. There were no differences between the groups in the terms of the C:D ratio and glaucoma severity.

### Surgical technique

All procedures were carried out under retrobulbar anaesthesia (2% xylocaine) by the same operator (J.K.). In the CS group, the eyeball was stabilized with 7–0 polypropylene traction (Prolene, Johnson & Johnson) through the peripheral part of the cornea; in the MS group, 6–0 silk traction (Peters Surgical) was used, suspending the superior rectus muscle. In both groups, a fornix-based conjunctival flap was dissected to expose the sclera. Then, the surgery was performed as described elsewhere^4^. Subsequently, phacoemulsification was performed through a corneal excision located 2.25 mm from the temple, using the phaco-chop technique and Megatron S4 HPS microsurgical system (Geuder, Heidelberg Germany), followed by the intracapsular implantation of the IOL. During each procedure, a calibrated timer was used to measure the operative time, i. e., the time between the administration of the retrobulbar anaesthesia and the removal of the eyelid retractor at the end of the surgery. The eyes were treated topically with postoperative steroids (Loteprednol, one drop three times daily for four weeks, and then tapered to b.i.d after a week), antibiotics (Moxifloksacinum, one drop three times daily for two weeks) and non-steroidal anti-inflammatory drugs (Bromfenak one drop two times daily for 4 weeks).

### Postoperative assessment

Control examinations were carried out before the procedure, on postoperative days 1 and 7, as well as 3, 6, and 12 months post-surgery. The protocol for each follow-up visit included a measurement of the IOP and BCVA, examination of the anterior chamber and fundus of the eye to monitor postoperative healing and potential complications. MRD, UEC, and SRMF were measured at 3, 6, and 12 months post-surgery^3^.

Additional interventions were carried out whenever there was an increase in IOP (≥ 16 mmHg) and evidence of a completely flat filtration bleb and subconjunctival fibrosis (evidence of congested and convoluted blood vessels above the scleral flap). Whenever fibrosis was detected based on the manifestations mentioned above, needling combined with subconjunctival injections of 5-FU (0.2 ml, 5 mg) was carried out. If no adverse events of this agent occurred, the injections were given for five consecutive days or until the resolution of fibrosis and IOP normalization. Suture lysis with an argon laser was performed during the first two weeks post-surgery if poor filtration through the bleb occurred due to too tight suturing of the scleral flap. Hypotonia was defined as an IOP ≤ 6 mmHg.

Surgical outcomes were analysed in terms of complete success and qualified success rates. Complete success was defined as maintaining an IOP ≤ 18 mmHg without glaucoma medications, and qualified success as the IOP ≤ 18 mmHg with no more than two glaucoma therapies. Surgical failure was defined as an IOP > 18 mmHg with more than two glaucoma medications or the need for reoperation. All glaucoma medications were discontinued from the day of the surgery. The therapy, following the EGS guidelines, was reintroduced whenever surgical treatment did not produce the target IOP.

### Statistical analysis

Statistical analyses were carried out using R, version 3.5.1. The normal distribution of quantitative variables was verified with the Shapiro–Wilk test, as well as according to the skew and kurtosis of the data and visual inspection of histograms. The homogeneity of variance was verified with Levene’s test. The Chi-squared test was used for between-group comparisons of nominal variables, whereas quantitative variables were compared with the Student’s *t*-test or Mann–Whitney U test depending on the distribution. The results recorded before the surgery and at the end of the 12-month follow-up were compared with Wilcoxon’s test for dependent variables. Additionally, mean/median differences between the study groups were calculated, along with their 95% confidence intervals. The probabilities of surgical success in the study groups were compared based on the survival curves. The results of all tests were considered significant at *p* < 0.05; all tests were two-sided. Data arepresented as mean ± standard deviation.

## Results

Phacotrabeculectomy with intraoperative eyeball fixation using traction suture at the corneal limbus and superior rectus muscle suturing was carried out in 23 and 18 patients, respectively. Demographic characteristics of the patients are shown in Table 1.

**Table 1:** Patient demographics.

Group		CS	MS	P*
*N*		23	18	–
Age (years)		72.39 ± 6.24	74.76 ± 7.78	0.309 *
Sex (female/male)		16/7	12/6	> 0.999
Operative time (mm.ss)		42.19 ± 9.94	42.63 ± 5.56	0.875
Glaucoma type	POAG	14	9	0.468
	PXG	7	9	
	Pigmentary	2	0	
LOCS III scale (NC_1_/NC_2_/NC_3_)		5/12/6	4/10/4	0.899

Data are presented as *n* or mean ± standard deviation; *CS* corneal suture, *MS* muscle suture, *POAG* primary open-angle glaucoma, *PXG* pseudoexfoliation glaucoma, *PG* pigmentary glaucoma, *LOCS III* Lens Opacities Classification System III.

*Student’s *t*-test or χ^2^ test.

### Intraocular pressure

In the CS group, the mean preoperative IOP was 23.6 ± 7.3 mmHg, which decreased by 38% at 12 months post-operation (15.2 ± 3.5 mmHg, *p* < 0.001). In the MS group, the mean IOP decreased by 39%, from 24.3 ± 6.6 mmHg preoperatively to 14.9 ± 3.5 mmHg at 12 months post-operation (*p* = 0.009). At 3 and 6 months postoperatively, the mean IOP was significantly lower in the CS group than in the MS group: 13.9 ± 3.0 mmHg vs. 17.7 ± 3.5 mmHg, and 13.9 ± 4.9 mmHg vs. 17.2 ± 3.5 mmHg, respectively (*MD* = 3.9, *CI*_*95*_ [1.7;6.1], *p* = 0.001, *p* = 0.005). However, the study groups did not differ significantly in terms of the IOP at the end of the 12-month follow-up (Table 2). The study was not powered to detect statistically significant changes in IOP.

**Table 2:** Statistical characteristics of the IOP (means, medians, standard deviations, and ranges) in the CS and MS groups at specific timepoints post-surgery.

Time	CS		MS		*MD* (95% *CI*)	*p
	Mean (*SD*)	Median (range)	Mean (*SD*)	Median (range)		
Pre-op	23.56 ± 7.35	22.00 (12.00; 39.00)	24.33 ± 6.61	23.00 (16.00; 40.00)	0.77 (− 3.65; 5.19)	0.726
3rd month	13.88 ± 3.00	14.00 (9.00; 19.00)	17.75 ± 3.48	17.50 (10.00; 25.00)	3.87 (1.68; 6.06)	0.001
6th month	13.89 ± 4.86	12.00 (9.00; 22.00)	17.24 ± 3.47	17.00 (11.00; 24.00)	2.05 (− 0.42; 4.51)	0.005
12th month	15.19 ± 3.47	15.00 (10.00; 22.00)	14.94 ± 3.55	15.50 (7.00; 21.00)	1.05 (− 2.44; 4.54)	0.541

*CS* corneal suture group, *IOP* intraocular pressure, *MS* muscle suture group, *SD* standard deviation, *Pre-op* pre-operatively, *MD* mean difference calculated as the result for the CS group minus the result for the MS group with the 95% confidence interval (CI).

*Student’s *t*-test.

### Glaucoma medications

Before the procedure, the mean number of antiglaucoma medications was 2.60 ± 0.99 and 3.00 ± 0.76 in the CS and MS groups, respectively. It decreased to 0.75 ± 1.06 and 0.33 ± 0.50 at 12 months post-surgery, respectively (*p* = 0.013, *p* = 0.004). No statistically significant between-group differences in the number of glaucoma medications were observed, either before or after the procedure (Table 3).

**Table 3:** Statistical characteristics of the number of glaucoma therapies (means, medians, standard deviations, and ranges) in the CS and MS groups at specific timepoints post-surgery.

Time	CS		MS		*MD* (95% *CI*)	*p
	Mean (*SD*)	Median (range)	Mean (*SD*)	Median (range)		
Pre-op	2.60 ± 0.99	3.00 (1.00; 4.00)	3.00 ± 0.76	3.00 (2.00; 4.00)	0.00 (− 1.00; 0.001)	0.420
12th month	0.75 ± 1.06	0.00 (0.00; 3.00)	0.33 ± 0.50	0.00 (0.00; 1.00)	0.00 (− 0.000007; 1.00)	0.456

*CS* corneal suture group, *MS* muscle suture group, *SD* standard deviation, *Pre-op* pre-operatively, *MD* median difference calculated as the result for the CS group minus the result for the MS group with the 95% confidence interval (CI).

*Mann–Whitney *U* test.

### Surgical success

**Figure 1:**
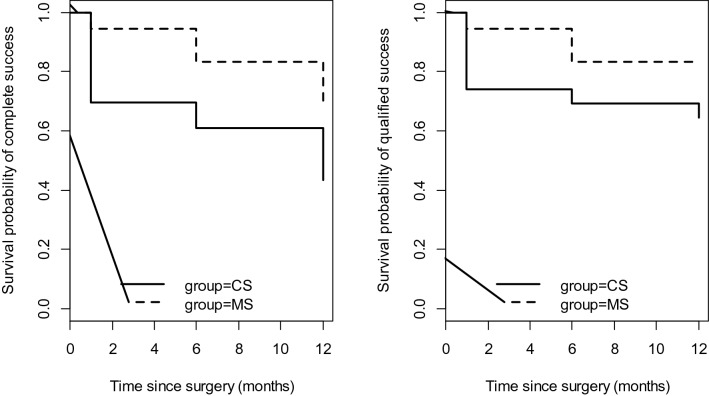
Survival curves illustrating the probability of complete success (IOP ≤ 18 mmHg without medications) and qualified success (IOP ≤ 18 mmHg with no more than two medications). *CS* corneal suture group, *IOP* intraocular pressure, *MS* muscle suture group.

In the CS and MS groups, complete success was achieved in 43.5% and 66.7% of patients, respectively (*p* = 0.100), and qualified success was achieved in 64.3% and 83.3% of patients, respectively (*p* = 0.200) (Fig. 1).

### BCVA

Before surgery, the mean BCVAs were 0.6 ± 0.2 and 0.4 ± 0.3, which improved to 0.78 ± 0.32 and 0.74 ± 0.3 at the end of the follow-up period in the CS and MS groups, respectively (*p* = 0.553)While no statistically significant between-group differences in the BCVA were observed preoperatively or at 3 and 12 months (*p* > 0.05), the mean BCVA at six months was significantly higher in the CS group than in the MS group (*MD* = 0.20, *CI*_*95*_ [0.0000003;0.30], *p* = 0.029) (Supplementary Table S1).

### Operative time

Mean operative times for the CS and MS group were 42.19 ± 9.94 min and 42.63 ± 5.56 min, respectively (*p* = 0.875).

### MRD

No between-group differences in the MRD were observed throughout the study period (Supplementary Table S2). Although 4 (17%) patients from the CS group and no patients from the MS group presented with post-operative ptosis at three months post-operatively, the between-group difference was not statistically significant (*p* = 0.118).

### UEC and superior rectus muscle function

Regardless of the analysed timepoint, the study groups did not differ significantly in terms of the UEC or SRMF values (Supplementary Tables S3 and S4).

### Complications and additional procedures

Subconjunctival injection of 5-FU was required in 8 (40%) and 6 (35%) patients from the CS and MS groups, respectively (*p* = 0.89). Needling was performed in 5 (25%) and 3 (17%) patients from the CS and MS groups, respectively (*p* = 0.75, Supplementary Table S5).

## Discussion

The present study demonstrated that while the type of intraoperative eyeball fixation during combined glaucoma and cataract surgeries was a determinant of short-term therapeutic success, it did not exert a significant effect on the IOP at the end of the 12-month follow-up. The IOPs in the CS group were lower than those in the MS group at both 3- and 6-months postoperatively. However, the between-group difference in the IOP at 12 months after surgery was not statistically significant. Moreover, patients from the CS group had a better BCVA than those from the MS group at the end of the short-term follow-up periods (3 and 6 months), but not at 12 months. However this study was not powered sufficiently to detect statistically significant changes between groups in efficacy.

In the study conducted by Li et al.^7^, patients from the corneal traction suture group presented with significantly lower IOP throughout the entire follow-up period (24 months). The between-group difference in the IOP was approximately 1–1.5 mmHg, but it needs to be emphasized that the study groups were relatively large (206 and 179 for the superior rectus traction suture and corneal traction suture groups, respectively), and hence, provided adequate statistical power for the calculations. According to Li et al., the between-group differences in the IOP were a consequence of a larger maximal bleb area in the corneal traction suture group than in the superior rectus traction suture group. Additionally, they found a difference in the number of cystic filtration blebs (13.1% in the superior rectus traction suture group vs. 6.7% in the corneal traction suture group). According to the authors of that study, the application of superior rectus traction suture might promote regeneration and scarring of the filtering bleb. Compression of the muscle might cause microhaemorrhages, and activation of fibroblasts during resorption of the blood might additionally promote scarring of the filtering bleb^13^. Furthermore, Li et al. observed that the filtering bleb in the corneal fixation group was more diffuse, which contributed to an incorrect position of the eyelid. Although we did not measure the filtering bleb in our patients, the lowest IOP values were found at 3 months post-surgery when the occurrence of ptosis was the highest; hence, also in the present study, an incorrect position of the upper eyelid might be associated with the presence of an elevated, diffuse filtering bleb.

Kaplan et al. suggested that grasping the superior rectus muscle with forceps causes inflammation, compression, congestion, and stasis in muscular arteries^9^. This, in turn, may contribute to the disruption of the intermuscular attachments between the levator and the superior rectus muscle, which eventually results in ptosis. However, this hypothesis was not confirmed in a retrospective study conducted by Patel et al.^12^.

Also, the results of our study do not seem to support the hypothesis mentioned above. We did not find statistically significant between-group differences in the MRD, UEC, and SRMF values. Furthermore, four cases of ptosis were recorded in the CS group; in three of these cases, ptosis resolved spontaneously within 6 months, but in one patient it persisted until the end of the follow-up period. Intriguingly, the patients from the CS group, who developed ptosis more frequently, presented with a lower IOP than the ptosis-free patients from the MS group at 3 months post-surgery.

According to Naruo-Tsuchisaka et al.^8^, post trabeculectomy ptosis developed in 19% of the patients who participated in their study. However, it is unclear which type of fixation was used by the authors and whether their patients received antimetabolite injections post-operatively. In the present study, the number of antimetabolite injections given in the CS and MS groups were 8 (40%) and 6 (35%), respectively (*p* = 0.89). The administration of wound modulating agents seems to exert an effect on the occurrence of postoperative ptosis.

In the study of Song’s et al.^11^, ptosis developed more often after combined glaucoma and cataract surgeries than after trabeculectomy alone (12.7% vs. 10.7%), which might be associated with different durations of the procedures. Similar results were also reported by Koh et al.^17^. In our study, the mean operative time did not differ significantly between the groups, and hence, was unlikely to affect the outcome of the treatment. Unfortunately, we did not find information on the operative times in the studies mentioned above. Furthermore, contrary to Song et al., we did not observe a levator dysfunction in the MS group^11^.

Surprisingly, visual acuity at 6 months was significantly better in the CS group than in the MS group. Trabeculectomy is known to be associated with an increased risk of the with-the-rule corneal astigmatism. An analysis of changes in corneal topography after glaucoma surgery demonstrated the mean change in astigmatic vector powers of 0.6 D; 30% of the study patients presented with transient astigmatism > 1 D^18^. Unfortunately, we did not analyse the corneal topography in our study; it is possible that this factor might explain the between-group difference in the BCVA.

Our study has some potential limitations. First, the follow-up duration was moderately long (12 months). Second, we did not analyse filtering bleb morphology, which is known to be associated with the occurrence of postoperative ptosis. However, no clear guidelines regarding bleb measurement have been developed so far, and the measurements were shown to have high intra-observer variance. Finally, the study groups were not enough large to to detect statistically significant differences in exploratory endpoints. Hence, further conclusions need to be derived from larger studies with longer follow-up durations.

In conclusion, this study demonstrated that the type of intraoperative eyeball fixation used during combined glaucoma and cataract surgeries exerted an effect on short-term therapeutic success, but did not modulate the IOP at the end of the 12-month follow-up period. At the end of the short-term follow-up (6 months), patients from the CS group presented with a significantly better BCVA than those from the MS group; however, no statistically significant between-group differences in the BCVA were found at 12 months post-surgery. At 3 months post-surgery, transient postoperative ptosis was observed only in four patients from the CS group (*p* = 0.058), which implies that the type of intraoperative eyeball fixation did not influence the occurrence of this complication; however, this hypothesis needs to be verified in a larger group of patients.

## Supplementary Information

Supplementary Information.

## Data Availability

The datasets generated and/or analysed during the current study are available from the corresponding authors on reasonable request; however, no information infringing on the privacy of the participants will be provided.
