# Supplementary material for: A prospective, randomised study of the effect of fixation sutures during phacotrabeculectomy on intraocular pressure and incidence of ptosis
Source: Sci Rep. 2021 Jan 12;11:548. doi: 10.1038/s41598-020-79635-x (PMC7804261; doi:10.1038/s41598-020-79635-x)
Supplement: Supplementary file 1 — Supplementary Information. [file 41598_2020_79635_MOESM1_ESM.pdf]

SUPPLEMENTARY MATERIALS

A prospective, randomised study of the effect of fixation sutures during phacotrabeculectomy on intraocular pressure and incidence of ptosis"

Joanna Konopińska[1,*], Łukasz Lisowski[1], Zofia Mariak[1], Małgorzata Wojnar[1], Iwona Obuchowska[1], Marek Rękas[2]

[1] Department of Ophthalmology, Medical University of Bialystok, Bialystok, Poland

[2] Department of Ophthalmology, Military Institute of Medicine, Warsaw, Poland

Supplementary Table S1. Statistical characteristics of the visual acuity (Snellen notification; means, medians, standard deviations, and ranges) in the CS and MS groups at specific timepoints post-surgery

**Time**	**CS**		**MS**		***MD* (95% *CI*)**	***p**
	**Mean (*SD*)**	**Median (range)**	**Mean (*SD*)**	**Median (range)**		
**Pre-op**	0.62±0.25	0.60 (0.10;1.00)	0.46±0.33	0.40 (0.05;1.00)	0.20 (-0.00003;0.40)	0.081
**3rd month**	0.83±0.19	0.90 (0.50;1.00)	0.72±0.33	0.80 (0.10;1.00)	0.10 (-0.10;0.20)	0.548
**6th month**	0.91±0.15	1.00 (0.50;1.00)	0.71±0.30	0.80 (0.10;1.00)	0.20 (0.0000003;0.30)	0.029
**12th month**	0.78±0.32	0.95 (0.10;1.00)	0.74±0.30	0.80 (0.10;1.00)	0.15 (-0.10;0.30)	0.553

CS: corneal suture group; MS: muscle suture group; *SD*: standard deviation; Pre-op: preoperatively; *MD*: median difference calculated as the result for the CS group minus the result for the MS group with the 95% confidence interval (CI); * Mann–Whitney U test.

Supplementary Table S2. Statistical characteristics of the MRD (means, medians, standard deviations, and ranges) in the CS and MS groups at specific timepoints post-surgery

**Time**	**CS**		**MS**		***MD* (95% *CI*)**	***p**
	**Mean (*SD*)**	**Median (range)**	**Mean (*SD*)**	**Median (range)**		
**Pre-op**	2.91±1.14	3.00 (1.00;6.00)	3.06±0.82	3.00 (1.50;4.50)	0.00 (-1.00; 0.50)	0.415

Time	CS		MS		*MD* (95% *CI*)	**p*
	**Mean (*SD*)**	**Median (range)**	**Mean (*SD*)**	**Median (range)**		
**3rd month**	2.87±1.45	3.00 (0.50;7.00)	3.15±0.95	3.00 (1.50;5.00)	0.00 (-1.00; 0.50)	0.400
**6th month**	3.09±1.16	3.00 (0.00;4.50)	3.13±0.89	3.00 (2.00;5.00)	0.00 (-0.50; 1.00)	0.711
**12th month**	2.88±0.62	3.00 (2.00;4.00)	3.33±1.09	3.00 (2.00;5.00)	0.00 (-1.50; 0.50)	0.350

CS: corneal suture group; MRD: marginal reflex difference; MS: muscle suture group; *SD*: standard deviation; Pre-op: preoperatively; *MD*: median difference calculated as the result for the CS group minus the result for the MS group with the 95% confidence interval (CI); * Mann–Whitney U test.

Supplementary Table S3. Statistical characteristics of the superior rectus muscle function (means, medians, standard deviations, and ranges) in the CS and MS groups at specific timepoints post-surgery

Time	CS		MS		*MD* (95% *CI*)	**p*
	**Mean (*SD*)**	**Median (range)**	**Mean (*SD*)**	**Median (range)**		
**Pre-op**	11.68±2.39	11.75 (7.00;17.00)	12.08±2.10	11.50 (9.00;16.00)	-0.40 (-1.85; 1.05)	0.580
**3rd month**	12.47±2.88	12.00 (9.00;20.00)	11.76±2.02	11.00 (9.00;15.00)	0.71 (-1.00; 2.42)	0.405
**6th month**	11.65±2.36	11.50 (6.50;15.00)	12.16±2.10	12.00 (9.00;16.00)	-0.51 (-2.10; 1.08)	0.518
**12th month**	13.56±3.19	13.50 (8.00;20.00)	12.56±1.76	12.00 (11.00;15.00)	1.00 (-1.04; 3.06)	0.320

CS: corneal suture group; MS: muscle suture group; *SD*: standard deviation; Pre-op: preoperatively; *MD*: mean difference calculated as the result for the CS group minus the result for the MS group with the 95% confidence interval (CI); *Student's *t*-test.

Supplementary Table S4. Statistical characteristics of the upper eyelid contour (means, medians, standard deviations, and ranges) in the CS and MS groups at specific timepoints post-surgery

Time	CS		MS		*MD* (95% *CI*)	**p*
	**Mean (*SD*)**	**Median (range)**	**Mean (*SD*)**	**Median (range)**		
**Pre-op**	7.70±1.48	8.00 (5.00;10.50)	8.17±1.35	8.00 (6.00;12.50)	-0.47 (-1.37; 0.44)	0.309
**3rd month**	7.71±1.59	7.00 (5.00;11.00)	7.29±1.64	7.50 (4.00;9.50)	0.42 (-0.68; 1.51)	0.445
**6th month**	8.00±1.48	8.00 (5.00;10.00)	7.81±1.52	8.00 (5.50;10.50)	0.19 (-0.88; 1.25)	0.722
**12th month**	8.09±0.78	8.00 (7.00;9.50)	7.89±1.56	8.00 (5.00;10.00)	0.20 (-0.75; 1.16)	0.663

CS: corneal suture group; MS: muscle suture group; *SD*: standard deviation; Pre-op: preoperatively; *MD*: mean difference calculated as the result for the CS group minus the result for the MS group with the 95% confidence interval (CI); *Student's *t*-test.

Supplementary Table S5. Postoperative complications

Postoperative Complications	CS, n (%)	MS, n (%)	**P*
**Leakage**	1 (5)	-	0.819
**Fibrosis**	8 (40)	6 (35)	0.753
**Anterior chamber cells**	2 (10)	3 (17)	0.752
**Hypotony**	1 (5)	-	0.819
**Posterior capsule opacification**	-	1 (6)	0.521
**Macular oedema**	1 (5)	-	0.532
**Shallow anterior chamber**	1 (5)	-	0.568

CS: corneal suture group; MS: muscle suture group; *χ^2 test
